# Antimullerian Hormone Changes after Laparoscopic Ovarian Cystectomy for Endometrioma Compared with the Nonovarian Conditions

**DOI:** 10.1155/2014/654856

**Published:** 2014-12-14

**Authors:** Chamnan Tanprasertkul, Sakol Manusook, Charintip Somprasit, Sophapun Ekarattanawong, Opas Sreshthaputra, Teraporn Vutyavanich

**Affiliations:** ^1^Department of Obstetrics and Gynaecology, Faculty of Medicine, Thammasat University, Pathum Thani 12120, Thailand; ^2^Center of Excellence in Applied Epidemiology, Thammasat University, Pathum Thani 12120, Thailand; ^3^Division of Physiology, Department of Preclinical Science, Faculty of Medicine, Thammasat University, Pathum Thani 12120, Thailand; ^4^Department of Obstetrics and Gynecology, Faculty of Medicine, Chiang Mai University, Chiang Mai 50000, Thailand

## Abstract

Laparoscopic ovarian cystectomy is recommended for surgical procedure of endometrioma. The negative impact on ovarian reserve following removal had been documented. Little evidence had been reported for nonovarian originated effects. *Objective.* To evaluate the impact of laparoscopic ovarian cystectomy for endometrioma on ovarian reserve, measured by serum antimullerian hormone (AMH), compared to nonovarian pelvic surgery. *Materials and Methods.* A prospective study was conducted. Women who underwent laparoscopic ovarian cystectomy (LOC) and laparoscopic nonovarian pelvic surgery (NOS) were recruited and followed up through 6 months. Clinical baseline data and AMH were evaluated. *Results.* 39 and 38 participants were enrolled in LOC and NOS groups, respectively. Baseline characteristics (age, weight, BMI, and height) and preoperative AMH level between 2 groups were not statistically different. After surgery, AMH of both groups decreased since the first week, at 1 month and at 3 months. However, as compared to the LOC group at 6 months after operation, the mean AMH of the NOS group had regained its value with a highly significant difference. *Conclusion.* This study demonstrated the negative impact of nonovarian or indirect effects of laparoscopic surgery to ovarian reserve. The possible mechanisms are necessary for more investigations.

## 1. Introduction

Endometriosis, the presence of endometrial tissue outside the lining of the uterine cavity, is one of the most common pelvic diseases in women. It is generally acknowledged that an estimated 6–10% of all women during their reproductive years are affected by this condition. In group of infertility women, 38 percent (20–50%) of them have endometriosis. If the patients have a history of chronic pelvic pain, the prevalence could be as high as 71–87 percent [1–4].

The ovarian endometriosis was recognized by the common term, namely, endometriotic cyst or endometrioma. The surgical intervention, laparoscopy, is the most useful option for further evaluation, treatment, and pathological removal [5]. Moreover, laparoscopic surgery is currently accepted as the procedure of choice for both diagnostic and therapeutic modalities. The systematic reviews showed that the excisional surgery or laparoscopic ovarian cystectomy for endometrioma provided more favorable outcomes than drainage and ablation surgery with regard to the recurrence of the endometrioma, recurrence of pain symptoms [6]. However, there were some reports that showed the negative impact on ovarian reserve, measured by serum antimullerian hormone (AMH) levels following ovarian cystectomy [7–11]. AMH levels represent the ovarian follicular pool and could be a useful marker of ovarian reserve. The clinical application of AMH measurement had been proposed in the prediction of quantitative and qualitative aspects in assisted reproductive technologies (ART). AMH seemed to be a better marker in predicting ovarian response to control ovarian stimulation than the patient's age, FSH (follicular stimulating hormone), estradiol, and inhibin B [12].

This negative effect had been explained by injury of adjacent ovarian follicles during the cyst wall excision. Also, the comparative study group of the most previous trials was benign, nonendometrioma ovarian cyst. To the best of our knowledge, there were a very few data which explored the possible effects of laparoscopic surgery and anesthesia on AMH in the nonovarian disease. The aim of current study was to evaluate the impact of laparoscopic ovarian cystectomy for endometrioma on ovarian reserve as measured by serum AMH, compared to nonovarian pelvic surgery.

## 2. Materials and Methods

This was a prospective cohort study which was conducted at Department of Obstetrics and Gynaecology in Thammasat University Hospital, Thailand. After approval from Ethical Institute Committee, the patients were enrolled with the following criteria; having 18–45 years; having regular menstrual cycles (21–35 days) at the time of operation; having no evidence of any other endocrine disorders such as diabetes mellitus, thyroid dysfunction, hyperprolactinemia, congenital adrenal hyperplasia, Cushing's syndrome, or adrenal insufficiency; undergoing laparoscopic ovarian cystectomy or laparoscopic nonovarian pelvic surgery for benign pelvic disease; having no previous history of adnexal surgery; having no suspicious findings of malignant ovarian diseases, never taking any medication such as oral pill and hormonal drugs within 3 months before the enrollment, pathological diagnosis of excised ovarian tissue confirmed it to be an endometriotic cyst in the study group and to consist of other benign pelvic diseases in control group. The participants were excluded if they had one of the following: polycystic ovarian syndrome according to the Rotterdam criteria [13] or operation conversion to exploratory laparotomy or pathological report as the malignant diseases.

All patients underwent the standard surgical procedures under general anesthesia. Each patient was appointed to visit the hospital on the seventh day and 1st, 3rd, and sixth months after laparoscopic ovarian cystectomy or nonovarian pelvic surgery. On each visit and preoperative day, blood samples would be obtained from the patients by venipuncture to measure the levels of AMH. The patient's sera were obtained from blood samples by centrifuge at 1400 ×g for 10 minutes to separate cellular contents and debris. The serum was transferred to sterile polypropylene tubes and stored at −70°C until assayed. Serum AMH levels were measured by enzyme-linked immunosorbent assay (ELISA, Diagnostic Systems Laboratories, Webster, TX, USA).

The sample size was calculated based on the determination of difference in means including confidence interval approach. The difference in means of serum AMH from previous studies was used for sample size calculation. From the study of Ercan et al. [11], mean preoperative AMH levels of the study and the control cases were 1.62 ± 1.09 and 2.06 ± 0.51 ng/mL, respectively. According to these values, the sample size was calculated by STATA program. The estimated number of women in each group was 40. In data and statistical analysis, descriptive statistics was used to describe study subjects' characteristics. Concentrations of serum AMH were interpreted between each sampling point (preoperative, postoperative first week, for 1st, 3rd and 6th months). The *P* value of less than 0.05 was considered as statistically significant.

## 3. Results

In this study, 90 women were enrolled. There were thirty-nine and 38 women in laparoscopic ovarian cystectomy (LOC) and laparoscopic nonovarian pelvic surgery (NOS), respectively, who had adequate complete data to analyze (Table 1). The mean age, weight, and height were not different between both groups. Duration of surgery and blood loss in LOC group were statistically significant in their differences from those of the NOS group. The mean diameter of endometrioma was 5.46 cm and ranged from 3 cm to 10 cm. Most of cases were unilateral but more so on the left side(58.97%). Bilateral disease was found to be only 15.38 percent. NOS group is composed of 19, 16, and 3 cases of laparoscopic hysterectomy (without adnexectomy), myomectomy, and adenomyomectomy, respectively.

**Figure 1:**
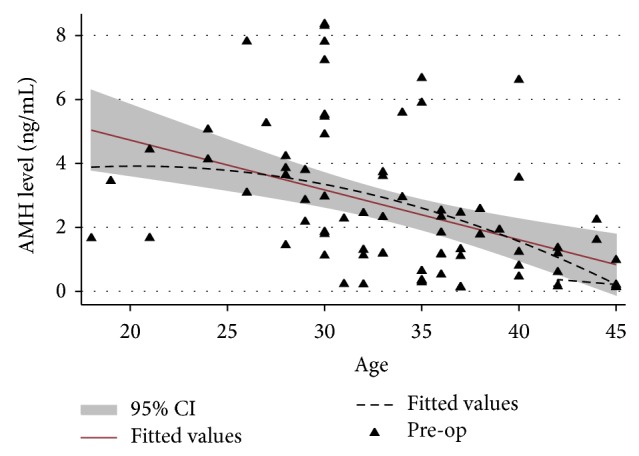
The correlation of serum AMH and age in participants. (AMH: antimullerian hormone).

As shown in Figure 1, the distribution of serum AMH levels was inversely correlated to the patients' age. After age of 40, the rate of declination was accelerated.

When comparing the serum AMH of LOC to NOS group, there was no statistically significant difference between groups at preoperative level, first visit on 7th day, second visit on first month, and third visit on 3rd month. However, there was statistically significant difference between groups at 6th month of operation. This negative change also occurred in the unilateral LOC group but there was no statistical difference.

## 4. Discussion

As shown in Figure 1, the AMH level was decreased with the advance of women age. This result demonstrated that ovarian reserve was declined throughout reproductive age. However, the cut-off value for serum AMH level for approving diminished ovarian reserve is still not determined. Previous research suggested that AMH was a promising marker [9]. But availability was limited because it was of high cost.

This study found that laparoscopic ovarian cystectomy in cases of endometrioma had negative effect on the ovarian capacity. Similar to previous studies which had been shown, the ovarian cystectomy can be harmful to ovarian reserve [7–10]. The present study showed that this adverse effect occurred immediately after operation and affected the patient for medium term, at least 6 months. In 9–12 months, we also investigated some patients; this diminished ovarian reserve effects still persist in most of them (data not available).

The results demonstrated that there is a strong negative impact of ovarian cystectomy on ovarian reserve; the guideline for management of ovarian cyst or endometrioma might be adjusted and reconsidered. Busacca et al. [14] reported that patients who underwent surgical operation for bilateral endometrioma had a prevalence of 2.4% ovarian failure immediately after surgery. This was consistent with Somigliana et al. [15] that in vitro fertilization (IVF) outcome and ovarian reserve were severely impaired in women who underwent operation for bilateral ovarian endometriomas. Similar to these findings, bilaterality is the major risk factor. Therefore, before ovarian surgery, not only morphological assessment but also careful ovarian function evaluation was needed. In case of low ovarian reserve, the surgical technique might be tailored and adjusted. The other alternative treatment may be a better option.

Most previous studies compared women who had ovarian cystectomy to the patients who had no history of surgery, for example, infertile women. Moreover, the study was cross-sectional design which did not have ability to demonstrate the causal relationship. The flaws of these were the uncertain causes of decline in ovarian reserve. Not only loss of follicles during stripping the endometriotic cyst but also blood loss in operative field could be another explainable reason. Atabekoğlu et al. [16] had reported the additional effect of total abdominal hysterectomy on serum AMH, 30% more loss of ovarian reserve. The surgery, hysterectomy, could reduce ovarian blood supply and resulted in temporary decline in ovarian reserve. In this study, we compared LOC to the NOS group in which laparoscopic surgery does not directly involve the ovaries, for example, hysterectomy and myomectomy. The postulated mechanism of decline in AMH especially in first 3 months might be due to the effect of blood loss and anaesthesia. This could be rescued by revascularisation of the ovaries.

**Figure 2:**
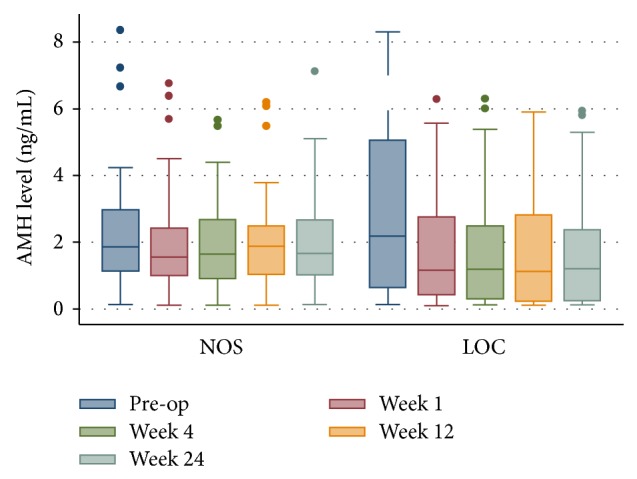
The changes in the serum AMH level at preoperative, post-op over periods of first week, 1, 3 and 6 months in the LOC and NOS groups. (AMH: antimullerian hormone, LOC: laparoscopic ovarian cystectomy, and NOS: nonovarian laparoscopic pelvic surgery).

As shown in Table 2 and Figure 2, AMH level of both groups had declined immediately after laparoscopic surgery. In LOC group the serum AMH level had declined until six months at least. However, this effect lasts only for short term, three months in NOS group. This might demonstrate that the effect of blood volume depletion includes the aesthetic impact during surgery. But it was only short term and temporary adverse effect.

There were some limitations of the study. Firstly, the ovarian reserve was measured by only single marker. Antral follicle count (AFC) is another useful marker for ovarian reserve. Sugita et al. [17] postulated the balancing effect of a healthy ovary which may compensate for a reduced ovarian reserve in the contralateral, affected ovary. Therefore, AFC may be a more accurate marker than AMH. However, this study did not have enough AFC data for analysis. Also, the measurement of AFC is subjective and evaluator-dependent. Secondly, the operations in the NOS group varied and were non-unique. Moreover, laparoscopic surgeons use a variety of techniques to operate on a case by case basis. Thirdly, there were some dropout participants, caused by loss follow-up and becoming pregnant.

## 5. Conclusion

Laparoscopic ovarian cystectomy in case of endometrioma had negative impact on the ovarian reserve, measured by serum AMH. This effect was sustained at least 6 months after operation. The negative impact occurred in patients who had nonovarian pelvic surgery but this adverse effect was only mild and temporary. This study showed the negative impact of nonovarian or indirect effects of laparoscopic surgery on ovarian reserve; however, the exact mechanisms were still unknown and needed to be explored more.

## Figures and Tables

**Table 1:** Characteristics of the participants between the laparoscopic ovarian cystectomy (LOC) and nonovarian surgery (NOS) group.

	LOC (*n* = 39)	NOS (*n* = 38)^*^	*P* value
Age (yrs)	32.74 ± 6.98	34.74 ± 5.2	0.16
Weight (kg)	52.51 ± 9.42	53.79 ± 6.93	0.49
Height (cm)	159.28 ± 4.63	156.86 ± 6.11	0.05
Duration of surgery (min)	67.05 ± 29.73	92.26 ± 34.20	0.001
Blood loss (mL)	61.15 ± 42.36	105.79 ± 57.50	0.002
Size of ovarian cyst (cm)	5.46 ± 1.70		
Bilateral	6 (15.38%)		
Stage of disease/rASRM score			
III	24 (61.54%)		
IV	15 (38.46%)		

rASRM: the revised American Society for Reproductive Medicine score.

^*^Laparoscopic NOS: 19 hysterectomies (without adnexectomy), 16 myomectomies, and 3 adenomyomectomies.

**Table 2:** Comparison of serum AMH level (ng/mL) between LOC and NOS group.

Serum AMH	LOC			NOS	Diff.	*P* value		
	Uni^*^ (*n* = 33)	Bi^*^ (*n* = 6)	All^*^ (*n* = 39)	(*n* = 38)	All/NOS	All/NOS	Uni/NOS	Bi/NOS
Preoperative	2.94 ± 2.47	2.01 ± 1.02	2.84 ± 2.47	2.33 ± 1.91	0.51	0.31	0.22	0.69
Postoperative								
7 days	1.71 ± 1.41	1.48 ± 1.07	1.76 ± 1.52	1.97 ± 1.64	−0.21	0.57	0.48	0.48
1 month	1.79 ± 1.74	1.41 ± 0.77	1.80 ± 1.70	2.24 ± 1.40	−0.44	0.22	0.23	0.16
3 months	1.86 ± 1.60	0.98 ± 0.42	1.72 ± 1.55	2.28 ± 1.46	−0.56	0.11	0.25	0.03
6 months	2.03 ± 1.74	0.94 ± 0.46	1.69 ± 1.63	2.44 ± 1.59	−0.75	0.04^§^	0.30	0.02^§^

Uni: unilateral, Bi: bilateral, and ^*^mean ± standard deviation.

^§^Statistically significant, Diff.: mean difference, and NOS: nonovarian laparoscopic pelvic surgery.

AMH: antimullerian hormone and LOC: laparoscopic ovarian cystectomy.
